# Clinical application of the CO_2_ laser in Ab externo Schlemm's canal surgery

**DOI:** 10.3389/fmed.2022.974056

**Published:** 2022-08-16

**Authors:** Liu Zhang, Yihua Yao, Qingxia Lin, Yanhong Li, Jianhui Zhang

**Affiliations:** ^1^Department of Glaucoma, Fuzhou Eye Hospital, Fuzhou, China; ^2^Department of Ophthalmology, The First Affiliated Hospital of Fujian Medical University, Fuzhou, China

**Keywords:** primary open-angle glaucoma, CO_2_ laser, complications, Schlemm's canal, success rate

## Abstract

**Purpose:**

This study aimed to investigate the clinical application of laser as a knife in Ab externo Schlemm's canal (SC) surgery and compare the efficacy and safety of the CO_2_ laser with the conventional procedure using a surgical knife.

**Methods:**

Patients who underwent either canaloplasty or trabeculotomy with CO_2_ laser system which was used to locate and ablate the outer wall of SC at the time interval between May 2020 and May 2021 were identified, their medical files were reviewed, and their results were compared with conventional surgery group who underwent canaloplasty or trabeculotomy with conventional surgical knife at the same time period. The following datas were conducted and compared: age, sex, intraocular pressure (IOP), number of drugs, best-corrected visual acuity (BCVA), mean deviation and pattern standard deviation of visual field examination, SC opening related complications.

**Results:**

A total of 49 patients (49 eyes) were included in this study, including 23 in the Laser surgery group and 26 in the conventional surgery group. Time for SC opening was 49.33 ± 25.23 s and 116.50 ± 31.79 s for laser surgery group and conventional surgery group, respectively. This difference between the two groups was statistically significant (*P* < 0.01). Hemorrhage occurred in five eyes during ablation for the laser surgery group and in 24 eyes for the conventional surgery group. In addition, anterior chamber penetration occurred in two cases for the laser surgery group and in six cases for the conventional surgery group. The success rate of identifying and opening outer wall of SC was 91.30% (21 eyes) for the laser surgery group and 76.92% (20 eyes) for the conventional surgery group. The difference between preoperative and postoperative intraocular pressure for each group was statistically significant (*P* < 0.01), and there were no statistically significant differences across the two groups in terms of postoperative IOP (*P* = 0.238) and BCVA (*P* = 0.389).

**Conclusion:**

Compared with the conventional procedure using a surgical knife, CO_2_ laser-assisted ablation of the outer wall of SC was less time-consuming and less technically challenging. CO_2_ laser-assisted ablation also resulted in fewer complications. Furthermore, it had a shorter learning curve and a higher success rate of identifying and opening SC.

## Introduction

As a critical link in the outflow pathway, normal Schlemm's canal (SC) is indispensable for maintaining normal intraocular pressure (IOP) (1). SC surgery primarily reduces intraocular pressure by increasing internal drainage. Compared with traditional anti-glaucoma surgery, it does not rely on filtering blebs to reduce IOP, with high patient comfort and easy postoperative management. SC surgery includes external and internal SC surgery. Ab externo SC surgery, including viscocanalostomy or canaloplasty, has been highlighted (2). As a bleb-independent surgical approach, it has shown good results in IOP reduction (3, 4). Precise location and correct dissection (SC outer wall) are important to achieve successful SC surgery, which requires a long learning curve, and such processes are technically challenging in conventional SC surgery using a surgical knife. The CO_2_ laser has been applied in glaucoma surgery and shown excellent efficacy and safety, for example, CO_2_ laser-assisted sclerectomy surgery (CLASS) (5). The CO_2_ laser is suitable for use in non-penetrating surgery because of its certain inherent characteristics: The laser effectively ablates the dry scleral tissue and is absorbed in water or aqueous solution within a short penetration depth (5). In this study, the CO_2_ laser-assisted system was used in ab externo SC surgery, and it achieved successful ablation of the SC outer wall, leading to satisfactory surgical results. Moreover, the clinical practice was summarized and reported.

## Methods

### Baseline information

In this retrospective study, 49 patients (49 eyes) with primary open-angle glaucoma hospitalized in Fuzhou Eye Hospital from May 2020 to May 2021 were enrolled. They were divided into laser surgery group (23 cases, 23 eyes) and conventional surgery group (26 cases, 26 eyes). In accordance with the tenets of the Declaration of Helsinki, all patients were informed of the operative procedures and related issues, and signed informed consent was obtained from the patients. This study received approval from the Institutional Review Board of the Fuzhou Eye Hospital in Fuzhou, China.

### Perioperative management

IOP was decreased pre-operatively as close to 21 mmHg as possible to mitigate the effect of high preoperative IOP on the collapse of the inner wall. IOP reduction was achieved by administering hypotensive eye drops or conducting anterior chamber paracentesis. Preoperative routine examinations were conducted, including best corrected visual acuity (BCVA), gonioscopy, ultrasound biomicroscopy (UBM), retinal nerve fibre layer (RNFL), mean deviation (MD) and pattern standard deviation (PSD) for the visual field examination. The examination results confirmed open-angle glaucoma. The surgeries were performed by a single experienced surgeon. Data were recorded, including the time needed to open the outer wall of the canal and the complications (such as hemorrhage and penetration) during the opening of the canal.

### Surgical approach

All surgeries were performed by a single experienced surgeon. Canaloplasty was applied in all surgeries firstly and then trabeculotomy was introduced as the alternative approach when the suture could not advance for 360 degrees. The CO_2_ laser-assisted system contained a beam micromanipulation system (OT-135P2, IOPtima Ltd., Israel) and a CO_2_ laser (SmartXide, DEKA, Italy). In the case of penetration, compound trabeculectomy was performed as an alternative. Success in identifying and opening the canal was indicated by the advancement of the blue prolene suture within SC using a gonioscope (regardless of the results of 360 degrees advancement). Details of the surgery are as follows.

#### Laser surgery group

**Figure 1:**
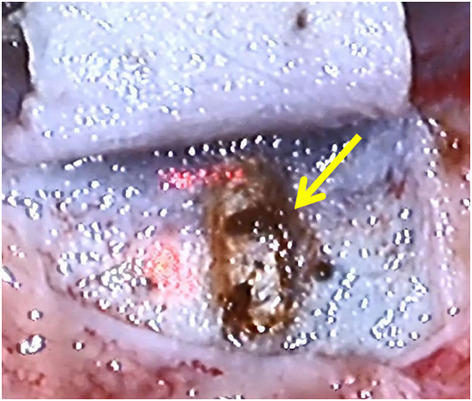
Laser-ablated outer wall of SC.

**Figure 2:**
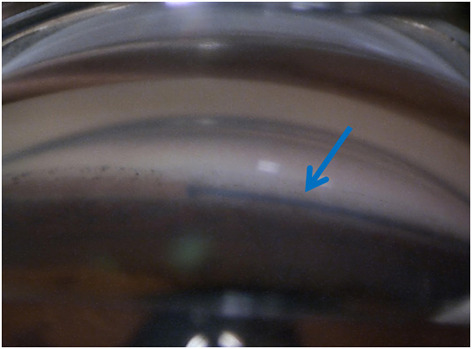
An advancing blue prolene suture was observed using gonioscope.

The patient lay in a supine position. The procedure was routinely sterilized, and a sterile surgical towel was laid out. After local anesthesia, the eyelid was opened. A limbal traction suture was placed to fixate the eyeball. A fornix-based conjunctival flap was created along the cornea limbus. Then a limbal-based flap measuring 4 × 4 mm with one-half scleral thickness was created, extended by 1-1.5mm into the clear cornea. A side cut was made at 10 o 'clock position with a side cutter to drain a small amount of aqueous humor. The CO_2_ laser system was employed. The laser beam, measuring 1 × 2 mm (length × width), conducted perpendicular ablation in the middle of corneoscleral junction until a channel structure and continuous aqueous humor percolation were observed, which marked the successful opening of the canal's outer wall and suggested that the ablation should be halted (Figure 1). A 6-0 prolene suture with a previously processed tip was inserted into SC from the surgical opening in a clockwise/counterclockwise fashion. An advancing blue prolene suture detected using gonioscope suggested the success of both identifying and opening the canal's outer wall (Figure 2). After the 360-degree journey in the canal was accomplished, the prolene suture came out from the other end of the canal. Next, a 10-0 polypropylene suture was tied to the tip of the 6-0 prolene suture, which would be withdrawn, pulling the 10-0 polypropylene suture into the canal. After the 6-0 prolene was completely retracted, the 10-0 polypropylene, which was kept within SC, was then tied to itself. The scleral flap was closed tight with 10-0 nylon sutures, and the conjunctival flap was closed with interrupted 10-0 nylon sutures.

#### Conventional surgery group

**Figure 3:**
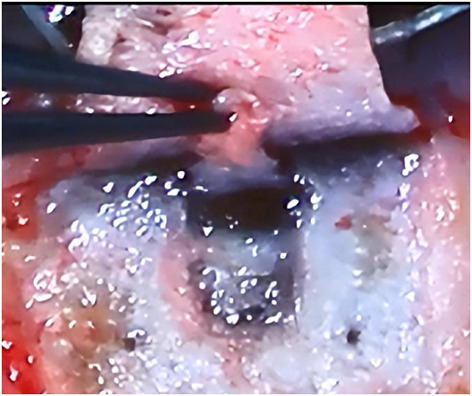
Manually dissected outer wall of SC.

The patient lay in a supine position. The procedure was routinely sterilized, and a sterile surgical towel was laid out. After local anesthesia, the eyelid was opened. A limbal traction suture was placed to fixate the eyeball. A fornix-based conjunctival flap was created along the cornea limbus. Then a limbal-based flap measuring 4 × 4 mm with one-half scleral thickness was created, and it was extended by 1–1.5mm into the clear cornea. A side cut was made at 10 o 'clock position with a side cutter to drain a small amount of aqueous humor. A deeper scleral flap (1.0 × 2.0 mm) was dissected under the superficial flap. It should be deep enough to enable the sight of the pigment of the choroidal tissue. In the process of extending it forward to the corneoscleral junction, SC was identified and the outer wall was excised (Figure 3). Aqueous percolation could be seen, suggesting that the outer wall of the canal was opened. The deep scleral flap was then excised. A 6-0 prolene suture with a blunted tip was inserted into SC from the surgical opening in a clockwise/counterclockwise fashion. The rest steps of the surgery were the same with those in the laser surgery group.

### Timing method

For the laser surgery group, timing started from the first laser shot by the CO_2_ laser-assisted system; for the conventional surgery group, timing started from the first cut when dissecting the deeper scleral flap. The terminal time was marked by a channel structure, and aqueous percolation was observed in the surgical field. Furthermore, timing in the laser surgery and conventional surgery groups was recorded.

### Statistical analysis

The cases which penetration occurred and resulted in failed opening of the SC would be excluded from the statistical analysis of the time required to locate and open the outer wall of the SC (hereinafter referred to as canal opening). Cases that will be included in postoperative statistical analysis of BCVA, IOP and anti-glaucoma medications should meet the following criteria: (1) the canal was successfully identified; (2) subsequent steps were successfully performed; (3) the 10-0 polypropylene suture was retained in the canal and tied to itself.

SPSS 25.0 (IBM, IL, USA) was used for statistical analysis. Descriptive results were presented as N (%) for categotical variables or mean (standard deviation) for continuous variables. Normality of the measurement data was evaluated using the Shapiro-wilk test. A paired t test was used to compare preoperative and postoperative values of parameters, for normally distributed differences in both groups, or via the paired Wilcoxon tests, in other cases. Independent samples t test was used for between-group comparisons (for normally distributed differences in both groups) or Wilcoxon Mann-Whitney tests (otherwise). Chi-square test (with Yates' corrected) or Fisher's exact test was used to compare between different percentages. Data were considered statistically significant when *P* < 0.05.

## Results

### Baseline information

The current study enrolled 49 patients (49 eyes), including 29 males (29 eyes) and 20 females (20 eyes). All 49 patients were corrected the information of the age, sex, preoperative IOP, and medications. However, only 41 patients were corrected the information of MD and PSD for the other eight patients can't finished the visual field examination due to the poor visual acuity. Results showed that the two groups had no statistically significant differences with regard to sex (Chi-squared test: *P* > 0.05), mean age (Wilcoxon Mann-Whitney tests: *P* > 0.05), preoperative IOP (Independent samples *t*-test: *P* > 0.05), medications (Wilcoxon Mann-Whitney tests: *P* > 0.05), MD (Independent samples *t*-test: *P* > 0.05) and PSD (Independent samples *t*-test: *P* > 0.05) (Table 1).

**Table 1:** Preoperative baseline information for both groups.

	**Laser surgery group** **(*****N*** = **23)** ***N*** **(%)**		**Conventional surgery group** **(*****N*** = **26)** ***N*** **(%)**		p*
**Sex**					
Male	14 (61%)		15 (58%)		0.821
Female	9 (39%)		11 (42%)		
**Laser surgery group**			**Conventional surgery group**			
	**Mean (SD)**	**Median** (P_25_, P_75_)	**Mean (SD)**	**Median** (P_25_, P_75_)	*p*
	(*N* = 23)		(*N* = 26)		
Age	60.30 (15.22)	63 (52, 73)	59.23 (13.86)	60 (52.5, 70.3)	0.703^†^
IOP (mmHg)	19.31 (6. 41)	17.7 (14.4, 25)	22.36 (7.27)	21.7 (15.9, 26.6)	0.128^&^
Medications	3.00 (0. 74)	3 (2, 4)	3.04 (0.82)	3 (2, 4)	0.856^†^
	(*N* = 19)		(*N* = 22)		
MD (dB)	−18.4 (8. 97)	−18.4 (−26.9, −9.8)	−18.2 (9.11)	−19.1 (−26.1, −10.7)	0.935^&^
PSD (dB)	7.23 (2. 98)	6.8 (5.2, 9.5)	8.56 (3.61)	9.1 (5.5, 11.8)	0.212^&^

*Chi-squared test; ^&^Independent samples t test [intraocular pressure (IOP) normally distributed in both groups].

*^†^Wilcoxon Mann-Whitney tests (age and drugs non-normally distributed in any group)*.

*SD, standard deviation; IOP, intraocular pressure. MD, mean deviation for the visual field examination; PSD, pattern standard deviation for the visual field examination*.

### Complications in opening SC

Based on our definition of success in identifying and opening the canal, the outer walls of the SC from 21 eyes in laser surgery group were all successfully opened. Of the 21 eyes, two encountered suture blockage, and trabeculotomy was introduced as an alternative approach. For the conventional surgery group, the outer walls of the SC from 20 eyes were successfully opened. Of the 20 eyes, three encountered suture blockage; thus, trabeculotomy was introduced as the alternative approach. The penetration rate in the laser group was lower than that in the manual group, but the Chi-square test with Yates' corrected showed that the difference was not statistically significant (*P* > 0.05, Table 2). Chi-square test (c) showed that statistically significant differences in intraoperative bleeding between the two groups (*P* < 0.01, Table 2). Severe complications, such as shallow anterior chamber, vitreous incarceration, explosive suprachoroidal hemorrhage and massive intraocular hemorrhage, did not occur during the surgery.

**Table 2:** Complications in identifying and opening SC for both groups.

	**Laser surgery group** **(*N* = 23)** ***N* (%)**	**Conventional surgery group** **(*N* = 26)** ***N* (%)**	***p***
• Hemorrhage (eyes) • Penetration (eyes)	• 5 (22%) • 2 (9%)	• 24 (92%) • 6 (23%)	• <0.001* • 0.331^‡^

*Chi-squared test; ^‡^Chi-square test with Yates'corrected.

### Time needed to open the outer wall of SC

Twenty-one cases (21 eyes) and 20 cases (20 eyes) were included for statistical analysis of the laser surgery and conventional surgery groups, respectively. Time to open the outer wall of SC for the laser surgery and conventional surgery groups was 49.33 ± 25.23 s and 116.50 ± 31.79 s, respectively. Wilcoxon Mann-Whitney tests showed that the difference in canal opening time between the two groups was statistically significant (*P* < 0.01, Table 3).

**Table 3:** Time needed to open SC for both groups. Excluding the cases wherein penetration occurred and resulted in failed identification of the canal.

	**Laser surgery group** **(*****N*** = **21)**		**Conventional surgery group** **(*****N*** = **20)**		***p* ^†^**
	**• Mean (SD)**	**Median** **(P_25_, P_75_)**	**• Mean (SD)**	**Median** **(P_25_, P_75_)**	
Time needed to open the canal (second)	49.33 (25.23)	40.0 (30.0, 65.0)	• 116.50 (31.79)	111.5 (93.3, 139.8)	<0.001

*†Wilcoxon Mann-Whitney tests (Time needed to open the canal non-normally distributed in any group)*.

### The postoperative statistical analysis included 19 cases (19 eyes) for the laser surgery group and 17 cases (17 eyes) for the manual surgery group

#### BCVA

Table 4 shows information about the BCVA before the operation and at 1 week after the operation. For the laser surgery group, 12 eyes (63.16%) showed stabilized or slightly improved BCVA at postoperative 1 week, whereas, seven eyes (36.84%) showed decreased BCVA. For the conventional surgery group, nine eyes (52.94%) had stabilized or slightly improved BCVA at postoperative 1 week, whereas eight eyes (47.06%) had decreased BCVA. No statistical difference was found between the BCVA prior to the operation and that at postoperative 1 week using Fisher's exact test. (*P* = 0.389).

**Table 4:** Pre-operative BCVA and BCVA at post-operative 1 week for both groups. Excluding the cases wherein compound trabeculectomy or trabeculotomy was performed as the alternative approach.

**BCVA**	**Laser surgery group** **(*N* = 19)** ***N* (%)**	**Conventional surgery group** **(*N* = 17)** ***N* (%)**	***p* ^↑^**
• Stabilised or slightly improved • At least one line lost	• 12 (63.16%) • 7 (36.84%)	• 9 (52.94%) • 8 (47.06%)	0.389

↑*Fisher's exact test*.

#### IOP

As shown in Table 5, the preoperative IOP for the laser surgery group and conventional surgery group was 18.63 ± 6.24 mmHg and 20.81 ± 6.42 mmHg, respectively, whereas the IOP at postoperative 1 week was 13.47 ± 2.83 mmHg and 14.72 ± 3.45 mmHg, respectively. Paired *t*-test showed that for each group, the difference between preoperative IOP and IOP at postoperative 1 week was statistically significant (*P* < 0.01). However, Independent samples *t*-test showed that the IOP of the laser surgery group and that of the conventional surgery group was not statistically significant at Preoperative and postoperative 1 week (*P* > 0.05), indicating that laser-assisted surgery had no negative effect on postoperative IOP reduction compared with the conventional procedure.

**Table 5:** Pre-operative values and values post-operative 1 week for both groups. Excluding the cases wherein compound trabeculectomy or trabeculotomy was performed as the alternative approach.

	**Laser surgery group** **(*****N*** = **19)**		**Conventional surgery group** **(*****N*** = **17)**		***P* ^↓^**
	**Mean (SD)**	**Median** **(P_25_, P_75_)**	**Mean (SD)**	**Median** **(P_25_, P_75_)**	
Pre-operative IOP (mmHg)	18.63 (6.24)	17.00 (13.3, 25)	20.81 (6.42)	20.2 (15.4, 24.5)	0.310^&^
Post-operative IOP (mmHg)	13.47 (2.83)	12.6 (11.3, 14.8)	14.72 (3.45)	13.6 (12.1, 18.4)	0.238^&^
*p* ^#^	0.001^•^		0.003^•^		
Pre-operative Medications	3.00 (0.75)	3.00 (2, 4)	3.24 (0.75)	3.00 (3, 4)	0.340^†^
Post-operative Medications	0.26 (0.65)	0 (0, 0)	0.76 (1.15)	0 (0, 2)	0.144^†^
*p* ^#^	0.004^▸^		0.001^▸^		

#*Intragroups analysis: ^•^A paired t test (drugs normally distributed in both groups), ▸Paired Wilcoxon tests (IOP non-normally distributed in any group)*.

*^↓^Intergroups analysis: ^&^Independent samples t-test (drugs normally distributed in both groups), ^†^Wilcoxon Mann-Whitney tests (IOP non-normally distributed in any group)*.

#### Anti-glaucoma medications

The number of anti-glaucoma medications before the surgery was 3.00 ± 0.75 (2–4 drugs) and 3.24 ± 0.75 (2–4 drugs) for the laser surgery and conventional surgery groups, respectively. In postoperative 1 week, the number of anti-glaucoma medications was 0.26 ± 0.65 (0–2 drugs) and 0.76 ± 1.15 (0–3 drugs), respectively. Paired Wilcoxon tests showed significant differences between the preoperative number of medications and medications in one post-operative week for both groups (*P* < 0.01). Wilcoxon Mann-Whitney tests results showed no significant differences in the number of administered medications in post-operative 1 week between the two groups (*P* = 0.144, Table 5).

## Discussion

As an anti-glaucoma surgery for reconstructing physiological aqueous outflow channel, external SC surgery has fewer complications than classical trabeculectomy because it does not rely on filtering blebs. External SC surgery, such as canaloplasty and trabeculotomy, has been proven to have a good IOP-lowering effect, and it is safe in patients with open-angle glaucoma (6–8). Nevertheless, many doctors are still reluctant to perform this kind of surgery because of its high technical requirements, and this surgery requires surgeons with relevant surgical experience. During surgical operation related to external SC surgery, locating and opening the outer wall of the SC are important. Locating and cutting the outer wall of the SC by manual dissection is the traditional way of opening the canal. However, mastering this surgical technique requires a long learning curve because of many factors such as the complexity of anatomical factors. In addition, experienced surgeons have high variability in whether they can successfully dissect the SC (3, 9, 10). Therefore, glaucoma doctors must find a tool to replace manual operation, simplify canal opening, improve the safety and effectiveness of SC opening and shorten the learning cycle of surgeons.

In China's medical market, CLASS surgical equipment (CO_2_ laser-assisted system), as a mature ophthalmic CO_2_ laser equipment, is increasingly developed. It uses a CO_2_ laser to ablate dry tissue and coagulate blood vessels, and the energy can be completely absorbed by a small amount of liquid. It repeatedly ablates deep scleral tissue in a controllable and standardized way, which shortens the learning cycle of manual doctors and improves the safety of surgery compared with classic non-penetrating deep sclerectomy (NPDS) (11–14). In recent 10 years, domestic and foreign researchers in CLASS surgery have also confirmed the feasibility of CO_2_ laser ablation of the deep sclera and the outer wall of the SC (15–18).

At present, little research is conducted on CO_2_ lasers in external SC surgery. We creatively applied the platform to external SC surgery and found that it is convenient to use in identification and opening the outer wall of SC, which can reduce the operation difficulty and shorten the operation time and learning curve of this kind of surgery. On the contrary, CLASS surgery is often parallel to the angle when ablating the outer wall of the SC. The size of the laser (3 × 1 mm) at the scleral margin is used to ensure an effective penetration area of a certain length, but the width of 1 mm has high requirements for positioning, and for some patients whose gray-blue junction is not evident or whose limbal structure is abnormal, the size of the laser may not be able to cover the outer wall of the SC. In the early stage of our research, the size of the laser spot is 3 × 3 mm; thus, the laser irradiation area can cover the SC. The outer wall of the SC can be ablated by careful separation. With rich surgical experience, the size of the laser spot gradually transits to 2 × 2 mm or 1 × 2 mm. We found that the 2 mm diameter is perpendicular to the corneoscleral edge, and the outer wall of the SC can also be dissected smoothly. This change can reduce the shallow scleral flap and show the impact on intraoperative astigmatism. The long-term results need to be followed up.

The canal opening time of the laser surgery group (49.33 ± 25.23 s) is significantly shorter than that of the conventional surgery group (116.50 ± 31.79 s). The main reason for the laser group to have a shorter canal opening time is the application of CO_2_ laser-assisted external wall ablation of SC, which does not need to manually dissect the deep scleral flap and locate the external wall of SC as the traditional operation, and the canal like structure after ablation in the laser group is obvious. Once the external wall is successfully ablated, aqueous humor exudation can be seen basically, with less intraoperative bleeding and clearer visual field Clear (see Figure 1 at the yellow arrow). In addition to bleeding disturbing the visual field, in the manual group, it is considered that the outer wall of the SC cannot be exposed due to the uneven depth of manual incision of the deep scleral flap, which cannot accurately reach the reliable depth of the incision. Therefore, the deep scleral flap needs to be dissected repeatedly. At the same time, if the outer wall is not fully opened, the adjacent tissue needs to be carefully removed under the deep scleral flap to be more satisfactory. The outer wall of the SC, and in this case, the removal of adjacent canal tissue, is easy to cause anterior chamber penetration, resulting in difficult subsequent steps. Compared with the laser group, it significantly increases the operation time and intraoperative operation load, and puts forward higher requirements for the operator's operation ability.

Hemorrhage occurred in only five cases (five eyes) for the laser surgery group and in 24 cases (24 eyes) for the conventional surgery group during canal opening time. The reason for the difference may be that the CO_2_ laser played a role in blood coagulation and thus reduced the incidence of hemorrhage (19).

Anterior chamber penetration occurred in two cases (two eyes) for the laser surgery group and in six cases (six eyes) for the conventional surgery group. The penetration rate of the laser group is only 8.7%, which is obviously lower than that of the manual group, but the Chi-square test with Yates' corrected showed that the difference was not statistically significant, which may be due to the small sample size, which needs to be further expanded in the future. This could be accounted for by the following reasons for the difference in the incidence of penetration. For one thing, it may be caused by a disadvantageous operative field as a result of hemorrhage, which resulted in a higher incidence of penetration. For another, after the ablation of the canal's outer wall was completed and the ablation came to a halt, the superfluous CO_2_ laser energy could be absorbed by the percolated humor (10). In this case, the internal wall of SC and the trabecular meshwork could be kept intact, reducing the incidence of penetration. However, ablation of the outer wall of the SC with the aid of the CO_2_ laser still has the risk of anterior chamber penetration. The reason for anterior chamber penetration in the laser group is analyzed. It may be that the marginal surface of the corneoscleral membrane to be ablated is not completely perpendicular to the laser beam, resulting in a slope like ablation. The ablation depth of tissue in the 1 × 2 mm area is unbalanced. Therefore, excessive penetration of front-end ablation into the anterior chamber occurs before aqueous humor exudation, so the intraoperative 1 × 2 mm is adjusted. There is no anterior chamber penetration after the surgical wound is perpendicular to the laser angle. Other researchers suggest that the laser energy can be appropriately reduced when deep ablation is performed near the outer wall of the SC (14); or the SC of some glaucoma patients has been blocked, and there is no liquid flow in the SC. Even if the outer wall of the SC is ablated, there may still be no liquid outflow. At this time, simple SC surgery may not be able to reduce IOP, so trabeculectomy can be considered directly.

There was no significant difference in BCVA, IOP and the number of anti-glaucoma drugs used between the two groups at 1 week after the operation. The average IOP at 1 week after operation in the laser group and the manual group decreased compared with the baseline IOP, and the difference was statistically significant. The range of IOP reduction in the laser group was lower than that in the manual group at 1 week after the operation, but there was no significant difference (27.70% and 29.26%, respectively), it may be related to the lower baseline IOP in the laser group than in the manual group. The mean IOP (14.72 ± 3.45) mmHg in the manual group 1 week after operation is similar to that reported by Ramesh s and Juliane matlach (the latter two are 13.7 ± 6.4 mmHg and 15.0 ± 6.7 mmHg, respectively) (20, 21).

The advantage of this study is to directly compare the application of the CO_2_ laser and traditional manual dissection in external SC surgery. To our knowledge, this is the first study to directly compare the effectiveness of the CO_2_ laser and manual dissection in locating and opening the outer wall of SC in glaucoma patients. External SC surgery requires high surgical technology and experience. If the surgical experience is insufficient or the technology is poor, and the operation is easy to fail due to the complexity of manual dissection or intraoperative bleeding, which reduces the operator's confidence in the operation and causes some difficulties for the development of this operation. With the assistance of CLASS platform laser, the most critical canal opening step in operation can be simplified, and the operation mode of the CO_2_ laser-assisted system is simple and easy to learn, which is beneficial to the doctor's operation experience and operation experience. The technical requirements are relatively lower, which is a good alternative to manual operation.

In conclusion, the application of the CO_2_ laser in external SC surgery has advantages over traditional manual sectioning and canal opening, which can improve the safety and effectiveness of such surgery and shorten the learning cycle of operators. Therefore, more ophthalmologists may be willing to choose to try external SC surgery, which will help promote the development of SC related surgery and provide a better basis for the treatment of glaucoma patients and doctors.

## Data availability statement

The raw data supporting the conclusions of this article will be made available by the authors, without undue reservation.

## Author contributions

Study concept and design, supervision, and critical revision of the manuscript: JZ, LZ, YY, QL, and YL. Data collection: LZ, QL, and YL. Analysis and interpretation of data, writing the manuscript, statistical expertise, and administrative, technical, or material support: JZ and LZ. All authors contributed to the article and approved the submitted version.

## Funding

Startup Fund for Scientific Research of Fujian Medical University (No. 2018QH1063); Natural Science Foundation of Fujian Province (No. 2020J05251).

## Conflict of interest

The authors declare that the research was conducted in the absence of any commercial or financial relationships that could be construed as a potential conflict of interest.

## Publisher's note

All claims expressed in this article are solely those of the authors and do not necessarily represent those of their affiliated organizations, or those of the publisher, the editors and the reviewers. Any product that may be evaluated in this article, or claim that may be made by its manufacturer, is not guaranteed or endorsed by the publisher.
