# Supplementary material for: Clinical application of the CO2 laser in Ab externo Schlemm's canal surgery
Source: Front Med (Lausanne). 2022 Aug 16;9:974056. doi: 10.3389/fmed.2022.974056 (PMC9424721; doi:10.3389/fmed.2022.974056)
Supplement: Supplementary file 3 [file Presentation_1.pdf]

Details of surgery

Laser surgery group: The patient lay in a supine position. The procedure was routinely sterilized, and a sterile surgical towel was laid out. After local anesthesia, the eyelid was opened. A limbal traction suture was placed to fixate the eyeball. A fornix-based conjunctival flap was created along the cornea limbus. Then a limbal-based flap measuring 4 x 4 mm with one-half scleral thickness was created, extended by 1-1.5mm into the clear cornea. A side cut was made at 10 o 'clock position with a side cutter to drain a small amount of aqueous humor. The CO2 laser system (OT-135-IOPtiMate) was employed. The laser beam, measuring 1 × 2 mm, conducted perpendicular ablation in the middle of corneoscleral junction until a channel structure and continuous aqueous humor percolation were observed, which marked the successful opening of the canal’s outer wall and suggested that the ablation should be halted（Fig. 1）. A 6-0 prolene suture with a previously processed tip was inserted into SC from the surgical opening in a clockwise/counterclockwise fashion. An advancing blue prolene suture detected using gonioscope suggested the success of both identifying and opening the canal’s outer wall（Fig. 2）. After the 360-degree journey in the canal was accomplished, the prolene suture came out from the other end of the canal. Next, a 10-0 polypropylene suture was tied to the tip of the 6-0 prolene suture, which would be withdrawn, pulling the 10-0 polypropylene suture into the canal. After the 6-0 prolene was completely retracted, the 10-0 polypropylene, which was kept within SC, was then tied to itself. The scleral flap was closed tight with 10-0 nylon sutures, and the conjunctival flap was closed with interrupted 10-0 nylon sutures.

Conventional surgery group： The patient lay in a supine position. The procedure was routinely sterilized, and a sterile surgical towel was laid out. After local anesthesia, the eyelid was opened. A limbal traction suture was placed to fixate the eyeball. A fornix-based conjunctival flap was created along the cornea limbus. Then a limbal-based flap measuring 4 x 4 mm with one-half scleral thickness was created, and it was extended by 1-1.5mm into the clear cornea. A side cut was made at 10 o 'clock position with a side cutter to drain a small amount of aqueous humor. A deeper scleral flap (1.0 × 2.0 mm) was dissected under the superficial flap. It should be deep enough to enable the sight of the pigment of the choroidal tissue. In the process of extending it forward to the corneoscleral junction, SC was identified and the outer wall was excised（Fig. 3）. Aqueous percolation could be seen, suggesting that the outer wall of the canal was opened. The deep scleral flap was then excised. A 6-0 prolene suture with a blunted tip was inserted into SC from the surgical opening in a clockwise/counterclockwise fashion. The rest steps of the surgery were the same with those in the laser surgery group.

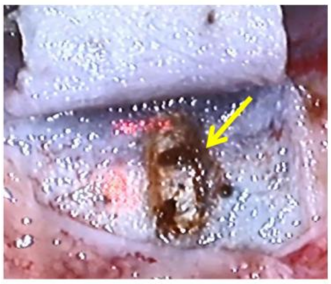

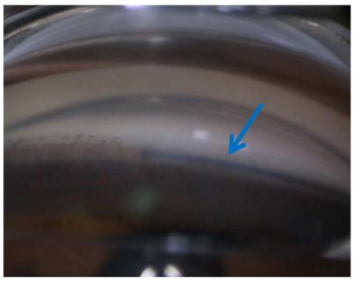

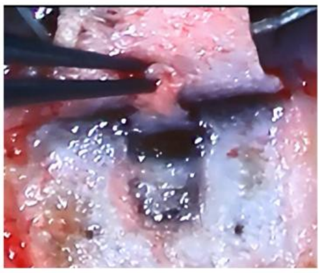

Fig. 1. Laser-ablated outer wall of SC.

Fig. 2. An advancing blue prolene suture was observed using gonioscope.

Fig. 3. Manually dissected outer wall of SC.
